# Structure of the Tubulin/FtsZ-Like Protein TubZ from *Pseudomonas* Bacteriophage ΦKZ

**DOI:** 10.1016/j.jmb.2013.03.019

**Published:** 2013-06-26

**Authors:** Christopher H.S. Aylett, Thierry Izoré, Linda A. Amos, Jan Löwe

**Affiliations:** MRC Laboratory of Molecular Biology, Francis Crick Avenue, Cambridge CB2 0QH, UK

**Keywords:** PDB, Protein Data Bank, EDTA, ethylenediaminetetraacetic acid, cytoskeletal, cytomotive, filamentous protein, X-ray crystallography, electron cryo-microscopy

## Abstract

*Pseudomonas* ΦKZ-like bacteriophages encode a group of related tubulin/FtsZ-like proteins believed to be essential for the correct centring of replicated bacteriophage virions within the bacterial host. In this study, we present crystal structures of the tubulin/FtsZ-like protein TubZ from *Pseudomonas* bacteriophage ΦKZ in both the monomeric and protofilament states, revealing that ΦKZ TubZ undergoes structural changes required to polymerise, forming a canonical tubulin/FtsZ-like protofilament. Combining our structures with previous work, we propose a polymerisation–depolymerisation cycle for the *Pseudomonas* bacteriophage subgroup of tubulin/FtsZ-like proteins. Electron cryo-microscopy of ΦKZ TubZ filaments polymerised *in vitro* implies a long-pitch helical arrangement for the constituent protofilaments. Intriguingly, this feature is shared by the other known subgroup of bacteriophage tubulin/FtsZ-like proteins from *Clostridium* species, which are thought to be involved in partitioning the genomes of bacteriophages adopting a pseudo-lysogenic life cycle.

## Graphical abstract

**Figure:**
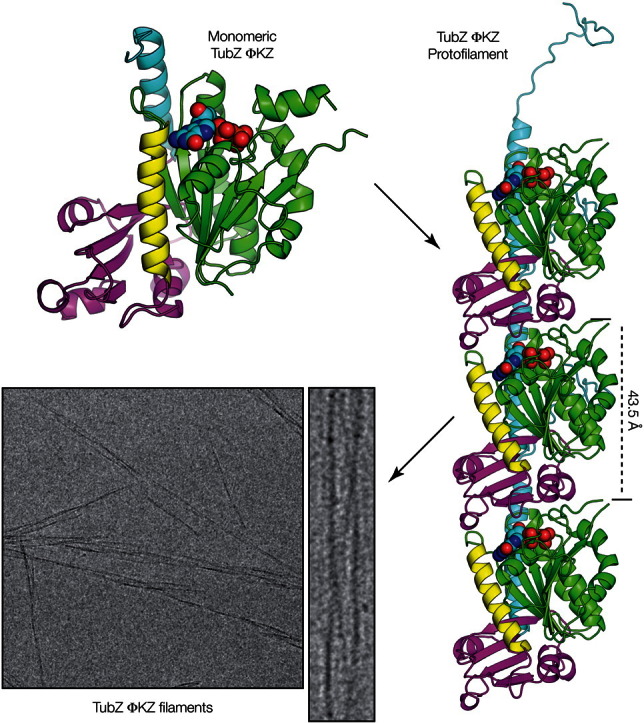

**Highlights:**

► Bacteriophage ΦKZ encodes a tubulin/FtsZ homologue, TubZ, similar to 201Φ2-1 PhuZ. ► We have resolved crystal structures of ΦKZ TubZ as both a monomer and a protofilament. ► ΦKZ TubZ undergoes structural changes only at its surface during polymerisation. ► Electron microscopy shows that ΦKZ TubZ forms intertwined helical filaments *in vitro*. ► This feature is common to other TubZs in *Bacillus* plasmids and *Clostridium* prophage.

## Introduction

Bacteriophages are believed to be the most abundant organisms in the world.^1^ Despite their number and diversity, the small size and simplicity of the majority of bacteriophages combined with their use of co-opted host proteins appears to have limited the requirement for their own cytomotive (formerly cytoskeletal) filament systems.^2,3^ Bacteriophage-encoded cytomotive filament proteins remain infrequent despite the number of sequenced genomes now available. However, although actin-like proteins have not yet been identified within a bacteriophage, both deviant Walker A cytoskeletal ATPases^4^ and tubulin/FtsZ family cytoskeletal proteins^5,6^ have been found.

Bacteriophage-encoded cytomotive filament proteins were first identified in the partitioning systems of pseudolysogenic bacteriophages. Such “temperate” bacteriophages do not cause immediate lysis of the host cell but are capable of maintaining themselves within the bacterial cytoplasm as a separate plasmid. This necessitates the presence of a bacteriophage-genome-encoded cytomotive filament to organise the accurate partitioning of the prophage into both daughter cells at cell division. The prototypical deviant Walker A, *parABS* plasmid partitioning system is encoded by *Escherichia coli* prophage P1^7^ and is responsible for the consistent segregation of the prophage as a plasmid. These systems were not immediately identified as parts of the bacterial cytoskeleton; however, later studies have shown that they may form filaments within the bacterial cell.^8–10^

Only two bacteriophage tubulin/FtsZ-like proteins have so far been reported. Each has been proposed to represent an exemplar of its own independent subgroup of bacteriophage tubulin/FtsZ-like proteins; however, the evolutionary relationships within bacteriophage tubulins remain ill-defined at this early juncture and will remain so until many further structures and functional data are available. In order to avoid prejudging the relationships between these proteins, we refer to both subgroups (and the newly discovered subject of this study) using the general name “TubZ” to denote tubulin/FtsZ-like proteins that “are different from, but related to both tubulin and FtsZ”.^5,6,11,12^

The first bacteriophage-encoded tubulin/FtsZ-family cytomotive filament protein to be reported was also a plasmid partitioning protein encoded by a pseudo-lysogenic bacteriophage (*Clostridium botulinum* pseudolysogenic prophage C-ST).^5^ Based on sequence and structure, it has been designated as belonging to the subgroup of TubZs believed to be responsible for accurate plasmid partitioning in a number of members of the *Bacillus* species.^11^ Congruently, the plasmids and bacteriophages involved are typically those upon which toxins are found, supporting a common history for these systems.^5^ Notably, both the C-ST and *Bacillus* TubZ proteins share the distinctive feature of polymerising to form double-helical twisted filaments, which have not been found in other members of the tubulin/FtsZ family of proteins.^5,12–14^ The exact biological role of TubZ from prophage C-ST is not known and hence its classification as a partitioning protein remains preliminary.

In contrast to the bacteriophage TubZ identified in *Clostridium*, the TubZ found within a *Pseudomonas* bacteriophage has not been proposed to be involved in partitioning of a pseudolysogenic prophage. *Pseudomonas* TubZ (which is also referred to as PhuZ) was first identified in bacteriophage 201Φ2-1 as a homologue of tubulin/FtsZ.^6^ While various close homologues have been found in other bacteriophages, it constitutes the only bacteriophage cytomotive filament protein for which no close plasmid or chromosomal homologues are known. Bacteriophage 201Φ2-1 is a member of the ΦKZ-like group of viruses,^15^ notable due to the size of their genome and coat.^16,17^ Although ΦKZ-like bacteriophages are known pseudolysogens,^18^ there is no evidence for a role in partitioning. 201Φ2-1 TubZ (PhuZ) is instead thought to localise bacteriophages within the bacterium, forming cytomotive filaments within the host cell that position virions at the cell centre for efficient release upon lysis.^6^

A previous crystal structure of 201Φ2-1 TubZ (PhuZ) revealed a protofilament-like arrangement with a weak longitudinal contact between adjacent subunits and exhibited an extended C-terminus running along the side of the protofilament bearing an acidic “knuckle”, which mutation revealed was critical for polymerisation.^6^ The difference between the active site from the 201Φ2-1 TubZ (PhuZ) structure and that found in canonical tubulin/FtsZ protofilaments posed the question of whether or not *Pseudomonas* TubZs function in the same way. Electron micrographs of polymerised 201Φ2-1 TubZ (PhuZ) suggested filaments consisting of flat ribbons of paired protofilaments, similar to those in the crystal structure.^6^

In this study, we looked at the protofilament contacts and filament structure formed by TubZ from *Pseudomonas* bacteriophage ΦKZ. We have solved the crystal structure of ΦKZ TubZ in the monomeric and polymeric forms, revealing structural changes in ΦKZ TubZ during polymerisation and showing that ΦKZ TubZ forms canonical tubulin/FtsZ subunit–subunit contacts and a functional tubulin/FtsZ active site. We propose that the three available structures of *Pseudomonas* TubZs (one from 201Φ2-1 and two from ΦKZ) describe a polymerisation–depolymerisation cycle. Electron cryo-microscopy of ΦKZ TubZ filaments polymerised *in vitro* implies a long-pitch twisted helical arrangement in which the constituent protofilaments are entwined around one another; different filament architectures could be observed, all showing twisted filaments. This is notably also a feature of filaments of the other known subgroup of bacteriophage-borne TubZs identified in *Clostridium*.^5^

## Results and Discussion

### Bacteriophage tubulin/FtsZ (TubZ) from *Pseudomonas* bacteriophage ΦKZ

**Fig. 1:**
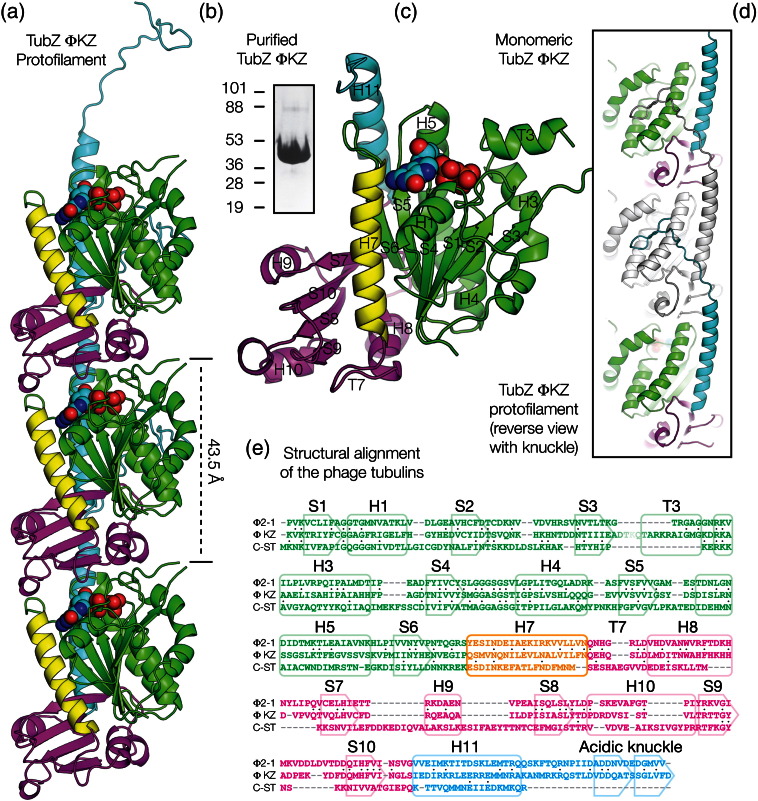
Monomeric and protofilament crystal structures of ΦKZ TubZ. (a) Cartoon representation of three subunits of the ΦKZ TubZ protofilament crystal structure; bound GDP shown as spheres. (b) Coomassie-stained SDS-PAGE of ΦKZ TubZ protein; molecular weight standards are expressed in kilodaltons. (c) Cartoon representation of the crystal structure of a monomer of ΦKZ TubZ annotated with the named tubulin/FtsZ secondary structural elements;^19^ bound GDP shown as spheres. (d) Cartoon representation of three subunits of the ΦKZ TubZ protofilament crystal structure; bound GDP shown as spheres, rotated 180° and with the central subunit coloured grey in order to highlight the C-terminal knuckle binding site. (e) Structural alignment of the three resolved bacteriophage tubulin/FtsZs. Colour scheme for all plates: green, GTPase domain; yellow, helix 7; magenta, activation domain; cyan, C-terminal helix; nucleotide in cyan and CPK colours.

In order to investigate the *Pseudomonas* group of bacteriophage tubulin/FtsZs, we synthesised the gene encoding TubZ from *Pseudomonas* bacteriophage ΦKZ (Taxon 169683; UniProt ID Q8SDC3), overproduced protein in *E. coli* and purified it to homogeneity (Fig. 1b). To gain a structural understanding of the protein, we began by crystallising and solving the structure of ΦKZ TubZ through molecular replacement. We solved the structure of crystals of ΦKZ TubZ to 2.0 Å (*R*/*R*_free_ = 0.17/0.21); the asymmetric unit contained a single subunit of the protein in a monomeric state (Fig. 1c; Table 1). The GTPase active site within the subunit was occupied by GDP, which was retained by ΦKZ TubZ through the purification process, and only the extreme N- and C-termini and a single region of surface loop T3 (residues 58–60) were not resolved within the electron density.

### ΦKZ TubZ adopts a tubulin/FtsZ fold

The crystal structures of ΦKZ TubZ revealed that it exhibits a tubulin/FtsZ protein family fold consisting of the canonical N-terminal GTP-binding domain, connected to the C-terminal GTPase activation domain through a conserved helix (H7) (Fig. 1c). Tubulin/FtsZ family proteins frequently possess N- and C-terminal extensions to the conserved core, and although ΦKZ TubZ has no significant N-terminal extensions to the canonical fold, it possesses a flexible C-terminal helix (H11) similar to that found in *Bacillus* TubZs, C-ST TubZ,^5^ and 201Φ2-1 TubZ (PhuZ),^6^ which tails into a long extended coil at around residue 300 (of 327) (Fig. 1a).

Comparison of ΦKZ TubZ to other members of the tubulin/FtsZ family of proteins reveals significant similarity to 201Φ2-1 TubZ (PhuZ), the proteins superimposing with a C^α^ RMSD of 2.4 Å (Fig. 1e). Given that both tubulin/FtsZs are found within the ΦKZ-like family of bacteriophages and that they share significant sequence identity (31%), this is not unexpected. Significantly, other tubulin/FtsZ family structures are more distantly related, including the other known phage tubulin, C-ST TubZ, the RMSD from these structures lying between 3 and 4 Å (C-ST TubZ, 3.8 Å, 16% sequence identity; in the phylogenetic tree presented by Kraemer *et al.*,^6^ C-ST corresponds to cst, ΦKZ to GP39, and 201Φ2-1 to GP59). Such segregation is compatible with the interpretation that the *Pseudomonas* TubZs may exist as an evolutionarily separate subgroup, perhaps one exclusive to the ΦKZ-like bacteriophages alone, although the sample number remains too small to be sure at this point.

### The surface of monomeric ΦKZ TubZ is incompatible with polymerisation

**Fig. 2:**
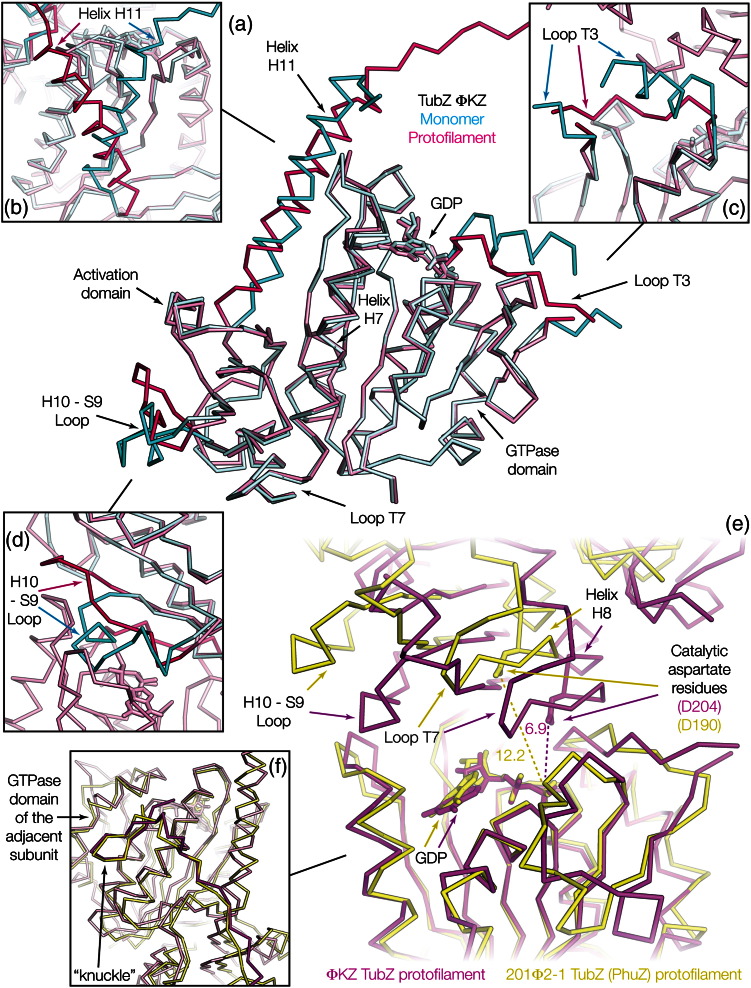
Conformational changes in ΦKZ TubZ during polymerisation. (a) Structural superimposition of the monomeric and protofilament crystal structures of ΦKZ TubZ. (b) Expansion showing the conformational changes undergone by helix 11 and the C-terminus on filament formation. (c) Expansion showing the conformational change undergone by loop T3 on filament formation. (d) Expansion showing the conformational changes undergone by the H10–S9 loop on filament formation. (e) Structural superimposition of the protofilaments of ΦKZ and 201Φ2-1 TubZ (PhuZ) comparing the subunit–subunit interface. (f) Structural superimposition of the C-terminal knuckle regions of ΦKZ and 201Φ2-1 TubZ (PhuZ). Colour scheme: cyan, monomeric ΦKZ TubZ; magenta/purple, ΦKZ TubZ protofilament; yellow, 201Φ2-1 TubZ (PhuZ) protofilament; coloured arrows denote the same region in different structures. All structures are C^α^ ribbons; distances are expressed in angströms.

The monomeric structure of ΦKZ TubZ exhibits several clear and interesting differences from those so far obtained for other tubulin/FtsZ family proteins. In terms of primary structure, there is a significant insertion within tubulin homology loop T3, which forms an α-helix instead of the conserved extended loop found in the majority of tubulin structures (Figs. 1c and 2a and c). Given the role of T3 in forming the subunit–subunit interface within a canonical protofilament, this would preclude formation of such a protofilament without significant rearrangement of the secondary structure. This is not the only such surface change, as the H10–S9 loop within the activation domain also lies in a conformation that would clash with an adjacent subunit (Fig. 2d) and the partially disordered C-terminal tail prominent in previous TubZ structures lies across the subunit interface, blocking polymerisation. This implied to us that either several conformational changes would be required for polymerisation into a canonical tubulin/FtsZ protofilament or that there might be differences in the protofilament.

### ΦKZ TubZ undergoes structural changes in order to form a protofilament

In order to discover how ΦKZ TubZ formed protofilaments, we crystallised and solved the structure of ΦKZ TubZ in the presence of the weakly hydrolysable GTP analogue GTPγS. The structure of a crystal form containing a crystallographic protofilament was obtained to 1.7 Å (*R*/*R*_free_ = 0.17/0.20) (Table 1). On examination, surprisingly, the GTPase active site was once again found to be occupied by GDP, not GTPγS, suggesting that nucleotide hydrolysis had occurred within the drop, possibly after forming GTPase-enabled protofilaments within the crystals. We discovered that the protein had undergone significant and intriguing changes in structure between the monomeric and filamentous crystal forms (Fig. 1a and d).

The structure of a protofilament of ΦKZ TubZ revealed that the monomer-to-protofilament transition substantially changes the conformation of the protein, returning all three aforementioned elements of the structure, which are inimical to the formation of a canonical tubulin/FtsZ-like protofilament to a similar state to that found in the majority of other tubulin and FtsZ homologues. This change entails the reorganisation of the C-terminal helix H11, and of both the T3 and H10–S9 surface loops, while both T3 and H10–S9 also lose their α-helical character, and would be essential for polymerisation (Fig. 2a–d).

A “curved–straight transition” reorienting the GTPase and activation domains through movement of H7 has been proposed during protofilament formation by tubulin and FtsZ; differences in domain orientation between the structures of related proteins in different states have provided some evidence for such movement in the case of tubulin while the structure of the *Staphylococcus aureus* FtsZ protofilament exhibits a different domain orientation to that observed in most FtsZ monomers.^20–22^ Significantly, in contrast to the surface changes required for protofilament formation, there was no evidence of any change in domain orientation for ΦKZ TubZ, the domains superimposing perfectly in both monomeric and protofilament crystal forms. This report represents only the second pair of such states for a single tubulin/FtsZ-like protein, *Bacillus thuringiensis* TubZ representing the first such pair.^14,23^ In both cases, no evidence for domain movement during polymerisation has been observed. The absence of such a transition for these two TubZs implies that such movements cannot be positively required for the polymerisation of tubulin/FtsZ-like proteins but cannot rule them out for other cases.

### ΦKZ TubZ forms canonical protofilaments with a competent GTPase site

**Fig. S1:**
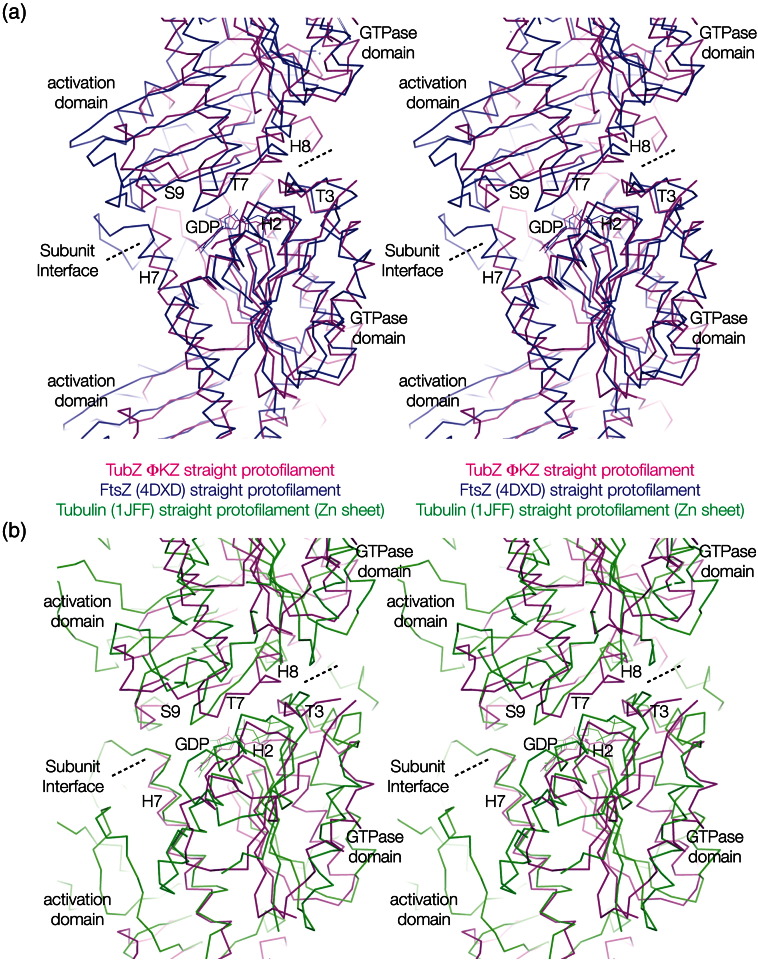
ΦKZ TubZ protofilaments compared to those of Tubulin and FtsZ. (a) Structural superimposition of the protofilament crystal structures of ΦKZ TubZ and FtsZ (PDB ID: 4DXD).^24^ (b) Structural superimposition of the protofilament crystal structures of ΦKZ TubZ and tubulin (PDB ID: 1JFF).^22^ Colour scheme: magenta, polymeric ΦKZ TubZ; blue, FtsZ protofilament; green, tubulin protofilament. All structures are shown as stereographic representations using C^α^ ribbons.

ΦKZ TubZ formed crystallographic protofilaments with a subunit–subunit interface essentially identical with that resolved for tubulin [Protein Data Bank (PDB) ID: 1JFF] and FtsZ (PDB ID: 4DXD).^22,24^ Superimposition of these three structures reveals that loop T7, the base of helix H8 and strand S9 occupy the same space in all three protofilaments (Fig. S1). The formation of a canonical tubulin/FtsZ protofilament explains the requirement for the structural changes observed between monomeric and polymeric states; the correct formation of the subunit–subunit interface is paramount for polymerisation, and it cannot otherwise take place.

The canonical tubulin/FtsZ subunit–subunit interface also resolves the question of how catalytic activity must occur in *Pseudomonas* TubZ protofilaments and implies that hydrolysis will weaken this interface in the same manner as in other tubulin/FtsZ-like proteins. Whereas the catalytic aspartate within loop T7 of 201Φ2-1 TubZ (PhuZ) is found 12.2 Å from the β-phosphate of GDP, too long a distance for efficient nucleotide hydrolysis, in protofilaments of tubulin (PDB ID: 1JFF) and FtsZ (PDB ID: 4DXD), this distance is 5.7 Å and 7.1 Å, respectively.^22,24^ The ΦKZ TubZ protofilament distance of 6.9 Å lies between the tubulin and FtsZ figures (Fig. 2e). The formation of a canonical protofilament substantially increases the surface area buried at the subunit interface. Whereas 201Φ2-1 TubZ (PhuZ) has an extremely small longitudinal surface contact (188 Å^2^ buried by both contributing subunits excluding the C-terminal tail), the ΦKZ interface encompasses 986 Å^2^ (buried by both contributing subunits excluding the C-terminal tail), a comparable figure to that of tubulin and FtsZ protofilaments (PDB IDs 1JFF and 4DXD1JFF4DXD bury 1666 and 1151 Å^2^, respectively).^22,24^ The acidic knuckle region at the C-terminus of ΦKZ TubZ becomes ordered in the protofilament, occupying the side of the adjacent subunit within the protofilament in the same location and orientation as found in 201Φ2-1 TubZ (PhuZ) and forming a similarly sized interface (1035 Å^2^ and 1027 Å^2^ are buried by both contributing subunits in the respective structures). This supports the proposed wider significance of this contact in these proteins and implies that this contact forms independently from the canonical subunit–subunit interface (Figs. 1d and 2f).^6^

It seems to us that the most likely interpretation is that the three *Pseudomonas* TubZ structures now available fortuitously represent different snapshots of the polymerisation–depolymerisation cycle of these proteins, the ΦKZ protofilament structure representing a tight polymerised protofilament complex, and the 201Φ2-1 structure representing a weaker protofilament encounter/departure complex. Combining structural information from both homologues, we propose that *Pseudomonas* TubZs may occupy a different conformation as a free monomer, surface loops being incorrectly ordered and the C-terminal helix and tail extending flexibly in solution as found in the monomeric ΦKZ structure. GTP exchange will favour formation of the active site, ordering surface loops and facilitating the rearrangement of T3 and H10–S9 so that a canonical tubulin/FtsZ subunit–subunit interface can form as the protein polymerises. The presence of adjacent subunits in the protofilament will then allow the C-terminal helix and tail to fold into the acidic knuckle, forming an extensive peptide interaction along the side of the protofilament, and substantially expanding the subunit–subunit interface. Stabilisation of the protofilament by the C-terminus may be important during hydrolysis. Once nucleotide hydrolysis has occurred, the canonical subunit–subunit interface dissolves, destabilising this interaction, but leaving the C-terminal tail still ordered on the surface of the filament, and allowing the protofilament to move between the canonical tubulin situation represented by the ΦKZ protofilament structure and a departure state similar to that of 201Φ2-1. This weakened protofilament state would collapse once the C-terminal tail becomes disordered, releasing a free monomer.

It is also possible that the weaker protofilament structure might represent an intermediate state in assembly. If GTP only favours organisation of the active-site loops, rather than fixing their conformation, a loose encounter complex with a longer inter-subunit spacing would result during the early stages of assembly. GTP would then be hydrolysed slowly within a protofilament due to subsequent rearrangement of T3 and H10–S9 to their active conformations. In the future, it will be important to investigate the exact role of the acidic knuckle in the polymerisation/depolymerisation process to discover whether it simply acts as a long, initial tether enhancing assembly and opposing disassembly, or whether it might also be directly involved in the regulation of GTPase activity.

### ΦKZ TubZ filaments are helical and composed of intertwined protofilaments

**Fig. 3:**
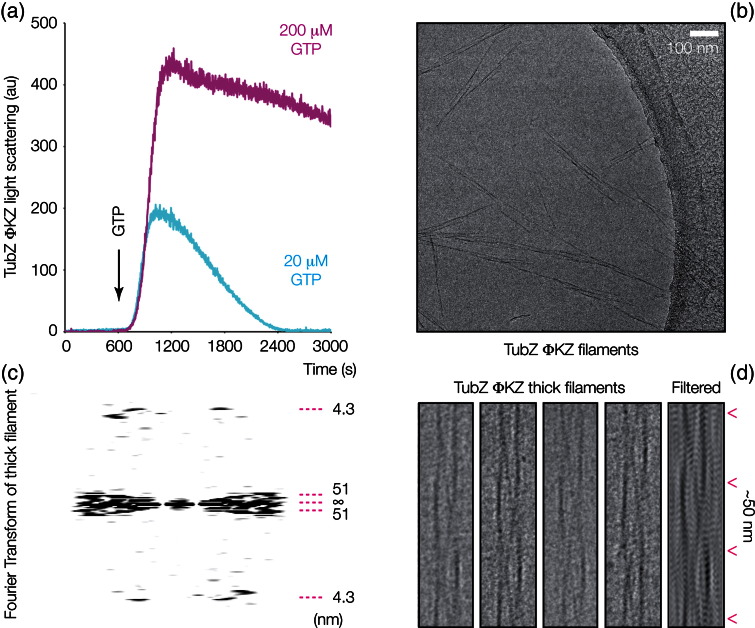
ΦKZ TubZ forms dynamic polymers from intertwined protofilaments. (a) Light scattering of ΦKZ TubZ on addition of GTP. Cyan trace indicates dynamic polymerisation and depolymerisation on addition of 20 μM GTP; magenta trace indicates polymerisation to a plateau on the addition of a saturating concentration of GTP (200 μM). (b) Electron cryo-microscopy of ΦKZ TubZ filaments with saturating concentrations of GTP, showing both polymerised bundles and separated filaments. (c) Fourier transform of a single thick ΦKZ TubZ filament; gyre and pitch layer lines are indicated alongside. (d) Electron micrograph of four single thick ΦKZ TubZ filaments, aligned to highlight the repeat and twist, adjacent to a filtered thick filament produced from the marked layer lines on its Fourier transform.

We went on to examine the polymerisation of ΦKZ TubZ in bulk through light scattering. ΦKZ TubZ filaments proved extremely dynamic in the presence of GTP; a 10-fold excess of GTP was insufficient for signal to reach a plateau; however, a hundred-fold excess was saturating, producing full polymerisation (Fig. 3a). Samples saturated with GTP were frozen in vitreous ice and viewed by electron cryo-microscopy, revealing that ΦKZ TubZ protofilaments combine to form a variety of filamentous structures *in vitro* (Fig. 3b). Thin filaments that appear to consist of several intertwined protofilaments predominate; however, larger structures are also present; some of these larger bundles appear to consist of multiple thin filaments that have coalesced, while others might possibly be fatter filaments consisting of larger numbers of protofilaments.

Separated filaments suitable for analysis were visible in electron micrographs (Fig. 3d); Fourier transformation of these filaments of ΦKZ TubZ indicates a subunit repeat of roughly 4.3 nm (by Fourier transform). This figure is in agreement with the figure for the protofilament crystal structure of ΦKZ TubZ (43.5 Å). We interpret this repeat length as implying that the filamentous state of ΦKZ TubZ is likely to be similar in nature to the canonical tubulin/FtsZ-like protofilament state found in our ΦKZ crystal structure. One small distortion relative to the crystal structure that must be present is clear, however. Whereas filaments of 201Φ2-1 TubZ (PhuZ) are currently believed to be formed by flat pairs of protofilaments adjacent to one another in a ribbon-like arrangement,^6^ a particular feature of ΦKZ TubZ filaments visible in electron micrographs was quite unexpected: there is a slight but visible twist to the protofilaments within the filament, which implies that the filaments form a helix with a long protofilament gyre length of ~ 51 nm (by Fourier transform). The constituent protofilaments are wrapped around one another, and it appears that there are at least three protofilaments in each bundle, as three separate traces can be clearly followed at each filament crossover. The width of the filaments (~ 15 nm) could, however, accommodate up to four or five protofilaments in theory. This is significant as the large distance required for each helical repeat (at least 153 nm for a three-filament bundle) explains clearly why a straight filament is possible in the crystals with very little distortion; the twist over each subunit interface will remain low.

The presence of twisted helical filaments of ΦKZ TubZ is interesting as it is only the third tubulin/FtsZ-like protein to form twisted filaments that has been identified; the others being the *Bacillus* TubZs^14^ and the other known bacteriophage tubulin/FtsZ, C-ST TubZ.^5^ Notably, this encompasses both TubZ subgroups identified within bacteriophages. One possible reason for these proteins to share such a feature is convergent evolution of cytomotive filament architectures; twist may be a favourable feature for tasks involving the movement of DNA or large cellular components, possibly acting as “rifling” to aid a linear direction of progress during polymerisation. A further possibility is shared ancestry; both proteins may have originated from a common ancestor in bacteriophages, which has adapted to carry out different tasks in pseudolysogenic prophages, some of which have become plasmids^5^ and large bacteriophages requiring virion centring.^6^

### Could features of the *Pseudomonas* and the *Bacillus* and *Clostridium* TubZs extend to one another?

The similarities we have identified between the two subgroups of TubZ proteins pose significant questions and hint at interesting possibilities. The *Bacillus* and *Clostridium* TubZs are believed to transport DNA within the cell; could this perhaps prove to be the case for *Pseudomonas* TubZs, the phage genome being localised prior to encapsulation? The C-terminal tail of *Bacillus*/*Clostridium* TubZs is also known to be involved in cofactor recruitment;^23^ however, that of *Pseudomonas* TubZs is clearly involved in polymerisation.^6^ Is it possible that both of these events occur in both proteins? This would provide an elegant manner in which a cofactor could affect the polymerisation of these proteins, through binding to the same site. Further work will be needed to unravel the mysteries these proteins present.

## Materials and Methods

### Sources

Unless stated, chromatography equipment was provided by GE Healthcare, chemicals were provided by Sigma Aldrich, crystallography consumables were provided by Hampton Research, and molecular graphics were generated using PyMOL (Schrödinger).

### DNA, genes, and vectors

The gene encoding *Pseudomonas* bacteriophage ΦKZ (Taxon ID 169683) TubZ (UniProt ID Q8SDC3) was synthesised codon optimised (GenScript, Hong Kong) in vector pET28a. Construct pET28a-ΦKZ-*tubZ* encoded the complete published sequence of ΦKZ TubZ without modifications and was used for light scattering and electron microscopy, whereas construct pET28a-ΦKZ-*tubZ*-His_6_ encoded an additional six histidine residues at the C-terminus and was used for crystallography.

### Expression

C41 *E. coli* (Invitrogen) carrying either of the two expression vectors were grown in 12 L of 2xYT broth. Cultures were grown at 37 °C and supplemented with 50 μg/L kanamycin, until reaching an optical density at 600 nm of 0.6. Expression was induced by the addition of a final concentration of 1 mM isopropyl-β-d-1-thiogalactopyranoside, and after expression overnight at 20 °C, the cells were harvested by centrifugation at 4***g***.

### Protein purification

The cell pellet from 12 L of culture was resuspended in 200 mL of 100 mM Tris–Cl and 500 mM NaCl, pH 8.0, and broken at 40 kPSI, 4 °C, using a cell disruption system (Constant Systems). Debris was removed by centrifugation at 45,000***g***. ΦKZ TubZ-His_6_ was retrieved by nickel affinity chromatography (5 mL HisTrap HP, 100 mM Tris–Cl, 500 mM NaCl, and 0–1 M imidazole gradient, pH 8.0), and crystallographic purity was achieved by ion exchange (1 mL HiTrap Q HP, 25 mM Tris–Cl, and 0–500 mM NaCl gradient, pH 8.0) followed by size-exclusion chromatography [HiLoad Sephacryl S200 16/60, 25 mM Tris–Cl, 200 mM NaCl, 1 mM ethylenediaminetetraacetic acid (EDTA), and 1 mM NaN_3_, pH 8.0].

Wild-type ΦKZ TubZ was retrieved by stepwise (10% sat. step) precipitation with saturated ammonium sulfate, pH 8.0. ΦKZ TubZ-containing pellets were pooled in 25 mM Tris–Cl, pH 8.0, and 1 mM EDTA, followed by purification by ion exchange (5 mL HiTrap Q HP, 25 mM Tris–Cl, and 0–500 mM NaCl, pH 8.0) and size-exclusion chromatography (HiLoad Sephacryl S200 16/60, 25 mM Tris–Cl, 200 mM NaCl, 1 mM EDTA, and 1 mM NaN_3_, pH 8.0).

### 90° Light scattering

Light-scattering experiments were performed using a Perkin Elmer LS55 Luminescence spectrometer in 25 mM Tris–Cl, 200 mM KCl, 5 mM MgCl_2_, and 0.5 mM EDTA, pH 8.0, at 25 °C with constant stirring of the 1-mL quartz cuvette. Excitation and emission wavelengths were both held at 400 nm, while the photon multiplier was set to 650 V. ΦKZ TubZ was added to a final concentration of 2 μM while either 20 or 200 μM GTP was added as indicated.

### Electron microscopy

Polymerised samples of ΦKZ TubZ produced as in light-scattering experiments (3 μL) were applied to glow-discharged holey carbon grids (Quantifoil R2/2 Cu/Rh 200 mesh; Agar Scientific) for 15 s, blotted and plunge-frozen in liquid ethane using a FEI Vitrobot. Grids were transferred to a FEI Polara G2 microscope operated at 300 kV. Images were acquired with defocus ranging from − 1 to − 3 μm on a back-thinned FEI Falcon 4k detector at 76,700 × nominal magnification, leading to a dose of 34 e^−^Å^− 2^, and processed using the MRC suite for electron microscopy.^25^ The magnification and pixel resolution of the microscope were calibrated using the molecular lattice of graphite before we undertook our experiment.

### Crystallography

Initial conditions were identified at the MRC-LMB crystallisation facility.^26^ ΦKZ TubZ-His_6_ crystals were produced in 500 nL to 500 nL protein to precipitant drops: the monomeric crystal form in 150 mM Tris–Cl, pH 8.0, 8.0% (v/v) ethylene glycol, and 20% (w/v) polyethylene glycol 5000 monomethyl ether, and the filamentous crystal form in 100 mM Na–citrate pH 5.5 and 20% (w/v) polyethylene glycol 3000. Artificial mother liquor supplemented to 25% (v/v) glycerol was used as a cryo-protectant. Diffraction from ΦKZ TubZ-His_6_ crystals was collected at European Synchrotron Research Facility beamline ID14eh1 and Diamond beamline I24. Data were processed with XDS,^27^ POINTLESS,^28^ and SCALA.^29^ Initial phases were determined by molecular replacement from PDB ID 3R4V using Phaser,^30^ and the model was built with MAIN^31^ and refined with REFMAC5^32^ and PHENIX.^33^

### Structural calculations and accession numbers

Structural superimpositions and alignments were carried out using the DALI-lite webserver.^34^ Surface area calculations were performed using the PDBe-PISA webserver.^35^ Coordinates and structure factors have been deposited in the PDB with accession numbers 3ZBP and 3ZBQ3ZBP3ZBQ for the monomeric and protofilament forms of ΦKZ TubZ, respectively.

## Author Contributions

C.H.S.A. and T.I. carried out all experiments. Experimental design, analysis, and manuscript preparation were performed by C.H.S.A., T.I., L.A.A., and J.L.

## Conflict of Interest Statement

The authors declare that they have no conflict of interest.

The following are the supplementary materials related to this article.

## Figures and Tables

**Table 1:** Crystallographic statistics for ΦKZ TubZ

	*Pseudomonas* phage ΦKZ–TubZ (monomer)	*Pseudomonas* phage ΦKZ–TubZ (protofilament)
*Protein*		
UniProt ID	Q8SDC3	Q8SDC3
Taxon ID	169683	169683

*Collection*		
Beamline	Diamond—I24	ESRF—ID14-1
Wavelength (Å)	0.9787	0.9334

*Crystal*		
Space group	*P*2_1_	*P*2_1_
Cell dimension		
*a*, *b*, *c* (Å)	41.5, 76.3, 54.1	43.5, 66.2, 52.6
β (°)	89.9	112.7

*Scaling*		
Resolution	2.0	1.7
Completeness (%)^a^	99.8 (99.5)	99.2 (95.2)
Multiplicity^a^	3.4 (3.3)	3.7 (2.9)
Half shell correlationa,b	0.991 (0.712)	0.998 (0.817)
(*I*)/σ(*I*)^a^	8.9 (2.6)	14.2 (1.7)
*R*_merge_^a^	0.124 (0.540)	0.075 (0.645)
*R*_pim_^a^	0.079 (0.348)	0.046 (0.453)
Wilson *B*-factor	17.9	17.4

*Refinement*		
*R*/*R*_free_^c^	0.1723/0.2103	0.1680/0.2032
Bond length rmsd (Å)	0.007	0.007
Bond angle rmsd (°)	1.126	1.010
Most favoured (%)^d^	98.3	98.7
Disallowed (%)^d^	0	0

*Deposition*		
PDB ID	3ZBP	3ZBQ

a Values in parentheses refer to the highest recorded resolution shell.

b Correlation coefficient between half sets, calculated from the intensities.

c Five percent of reflections were randomly selected before refinement in order to calculate *R*_free_.

d Percentage of residues lying in the chosen areas of the Ramachandran plot (PROCHECK).
